# Efficacy and safety of the neoadjuvant chemoimmunotherapy with pembrolizumab plus docetaxel and cisplatin in resectable locally advanced squamous cell carcinoma of the head and neck

**DOI:** 10.3389/fimmu.2025.1664892

**Published:** 2025-12-17

**Authors:** Jing Wang, Wenqian Zhang, Dongning Huang, Zhu Liu, Wei Zhang, Zhendong Li

**Affiliations:** 1 Department of Head and Neck Surgery, Cancer Hospital of China Medical University/Liaoning Cancer Hospital and Institute, Shenyang, China; 2 Department of Biostatistics, School of Public Health, Fudan University, Shanghai, China

**Keywords:** neoadjuvant, chemotherapy, immunotherapy, head and neck cancer, combined positive score

## Abstract

**Background:**

Head and neck squamous cell carcinoma (HNSCC) is the most common type of tumor originating from the squamous epithelium of the oral cavity, oropharynx, larynx and hypopharynx. There is an urgent need to enhance therapeutic efficacy and ensure the function preservation of HNSCC treatment. This study aimed to evaluate the efficacy and safety of neoadjuvant chemoimmunotherapy with pembrolizumab plus docetaxel and cisplatin in patients with resectable locally advanced HNSCC.

**Methods:**

This was a prospective single-center, single-arm, open-label, phase II study involving patients with locally advanced HNSCC who were treated with pembrolizumab (200 mg) plus docetaxel (50 mg/m^2^) and cisplatin (50 mg/m^2^). After two cycles of the neoadjuvant chemoimmunotherapy, patients either proceeded to surgery or refused surgery and continued with chemoimmunotherapy and/or chemoradiotherapy. The primary endpoint was the objective response rate (ORR), while the second endpoints were primary lesion complete pathologic response (pCR), progression-free survival (PFS) rate, overall survival (OS) rate and safety.

**Results:**

Overall, 52 patients with HNSCC were enrolled (45 males/7 females), with a median age of 61 years. The ORR rate was 87.8% (95% confidence interval [CI]: 75.2–95.4). Of the 27 patients who underwent resection, 12 (44.4%, 95% CI: 25.5–64.7) achieved a pCR and 15 (55.6%, 95% CI: 35.3–74.5) achieved an MPR. For the overall population, the PFS rates were 89.8% (95% CI: 77.2–95.6), 87.5% (95% CI: 74.2–94.2), and 72.7% (95% CI: 50.6–86.1), and the OS rates were 95.9% (95% CI: 84.7–99.0), 93.6% (95% CI: 81.5–97.9) and 84.9% (65.0–93.9), at 6 months, 12-months and 18 months, respectively. The most common adverse events were nausea/vomiting (12.2%), fatigue (10.2%), rash (8.2%), neurotoxicity (8.2%) and aminotransferases elevation (8.2%). Grades 3 and 4 adverse events occurred in 12 (24.5%) and 2 (4.1%) patients, respectively.

**Conclusions:**

Pembrolizumab plus docetaxel and cisplatin reveals encouraging survival benefit with a manageable safety profile in patients with HNSCC.

## Highlights

- Neoadjuvant chemoimmunotherapy has been regarded as a promising treatment option for patients with resectable locally advanced HNSCC.
- Neoadjuvant chemoimmunotherapy with pembrolizumab plus docetaxel and cisplatin presented encouraging efficacy regarding radiographic and pathological anti-tumor response and an acceptable safety profile.
- This study provides further guidance and assurance of using pembrolizumab plus docetaxel and cisplatin to treat patients with resectable locally advanced HNSCC.

## Introduction

Head and neck cancer represents a type of cancer that is commonly diagnosed worldwide, accounting for over 700,000 new cancer cases and 350,000 cancer - related deaths globally each year (1). Head and neck squamous cell carcinoma (HNSCC) is the most common type of cancer in the head and neck region, which serves critical physiological functions. Either from a direct consequence of the tumor invasion or due to the cancer treatment, patients with HNSCC frequently suffer from damage to these important functions, which exerts a substantial detrimental influence on their health-related quality of life (HR-QoL). Therefore, there is an urgent need to improve both the therapeutic efficacy and ensure the function preservation and acceptable HR QoL of HNSCC treatment.

As expected, traditional surgical approaches to HNSCC may lead to significant cosmetic and functional morbidity, and the extent varies case by case. Multidisciplinary treatments, integrating surgery, chemotherapy and radiation, have been investigated with the aim of maximizing therapeutic outcomes in recent years. Nevertheless, these approaches continue to exert a considerable impact on patients’ functional status (2). Despite these treatments, patients with HNSCC still have a high risk of local recurrence and distant metastasis, with an approximately 50% overall survival (OS) rate at 5 years (3).

Neoadjuvant chemoimmunotherapy has been regarded as a promising therapeutic option for patients with resectable locally advanced HNSCC (2, 4). Although this treatment has not been fully investigated, it has the potential to offer benefits of organ preservation and reduction of local and/or regional recurrence. Furthermore, programmed cell death protein 1 (PD-1) inhibitors, such as nivolumab and pembrolizumab, have been approved for treating recurrent and metastatic HNSCC (5–7). Previous studies have shown that chemotherapy can upregulate PD-L1 expression on tumor cells, thereby enhancing their susceptibility to subsequent PD-1 blockade immunotherapy (8, 9).

Pembrolizumab is a PD-1 blocker that minimizes the inhibitory effects of PD-1 on T cells, thereby enhancing the anti-tumor action of T cells. The combination of pembrolizumab and chemotherapy has become the standard first-line treatment for recurrent or metastatic HNSCC. However, existing data on pembrolizumab in locally advanced HNSCC mainly focus on monotherapy, and evidence regarding its neoadjuvant use remains limited. The newly released results from the phase 3, open-label, randomized controlled KEYNOTE-689 trial, which centered on patients diagnosed with locally advanced HNSCC, set EFS as its primary efficacy endpoint. The findings revealed that the combination of neoadjuvant and adjuvant pembrolizumab with standard therapy achieved markedly superior EFS compared with the control group, with a low incidence rate of adverse events (10). Nevertheless, it is worth noting that this trial did not include a comparative analysis of a neoadjuvant chemoimmunotherapy regimen that combines pembrolizumab with docetaxel and cisplatin.

Therefore, we conducted a prospective, single-center, single-arm, open-label, phase II study to explore the efficacy and safety of pembrolizumab combined with chemotherapy as a neoadjuvant treatment in patients with resectable locally advanced HNSCC.

## Methods

### Study design and patients

This was a prospective, single-center, single-arm, open-label, phase II clinical study conducted at Liaoning Cancer Hospital and Institute (Shenyang, China) between June 2022 and April 2024. Eligible patients were ≥18 years old with histologically confirmed, previously untreated, resectable, locally advanced HNSCC (stage II–IVB) of the oral cavity, oropharynx, hypopharynx, or larynx. Stage II hypopharyngeal patients were included prospectively because their tumors are often adjacent to the esophagus, and upfront surgery could require total laryngectomy, causing severe functional impairment; multidisciplinary assessment confirmed that these high-risk patients were suitable for neoadjuvant chemoimmunotherapy with pembrolizumab plus docetaxel and cisplatin. Other inclusion criteria included an ECOG performance status of 0–1, at least one measurable lesion per RECIST v1.1, and adequate organ function (absolute neutrophil count ≥1,000/mcL; platelet ≥75,000/mcL; hemoglobin ≥8 g/dL; no erythropoietin dependency within 7 days after screening; serum creatinine ≤1.5 × ULN or creatinine clearance ≥60 mL/min; total bilirubin ≤1.5 × ULN or direct bilirubin ≤ULN; for patients with liver metastasis, AST ≤2.5 × ULN and ALT ≤5 × ULN; plasma proteins >2.5 mg/dL; INR or PT ≤1.5 × ULN). Female participants were required to use contraception and not be pregnant or breastfeeding; male participants were also required to use contraception during the study and for 6 months afterward. Exclusion criteria included prior anti-PD-1 therapy and a history of other malignancies within the past 5 years, except for treated stage I/II squamous cell carcinomas or *in situ* cancers.

### Treatment regimen

After an evaluation using the combined positive score (CPS), the patients were treated with neoadjuvant pembrolizumab (200 mg) plus docetaxel (50 mg/m²) and cisplatin (50 mg/m²) on day 1 of each 3-week cycle, for a total of two cycles. Treatment was discontinued at the investigator’s discretion if there was unacceptable toxic effects or disease progression. Two weeks after completing the two-cycle treatment, an evaluation combining immunohistochemistry and imaging analyses was conducted, along with discussions by a multidisciplinary team comprising surgeons, medical oncologists, and radiologists to guide subsequent treatment. Based on the response received from the neoadjuvant therapy and tumor burden, the multidisciplinary team discussed the treatment plan with patients and their families and caregivers. If the regression was not evident or residual tumor was suspected, resection was performed according to the originally planned surgical margins. If significant tumor regression was observed, the surgeon could reduce the resection scope to the outer margin of the regressed tumor. During the operative tumor resection procedure, tissue samples from at least four directional margins were sent for frozen section analysis, and the surgical procedure was completed only after all frozen section results from these margins confirmed no cancer cell was detected. Patients who refused surgery received chemoimmunotherapy and/or chemoradiotherapy, while the remaining patients underwent relevant surgery; no later than 6-week post-surgery (11, 12), patients who achieved an MPR received chemoimmunotherapy and chemoradiotherapy, and patients who achieved a pCR received chemoimmunotherapy.

Radiographic assessment using contrast-enhanced computed tomography was performed before neoadjuvant treatment with pembrolizumab, docetaxel, and cisplatin, as well as two weeks after treatment and prior to surgery, in accordance with the Response Evaluation Criteria in Solid Tumors (RECIST) version 1.1. The programmed death-ligand 1 (PD-L1) expressions in the primary lesions before the neoadjuvant therapy and in the residual lesions were assessed by using the 22C3 assay. To explore the correlation between PD-L1 expression and the efficacy of neoadjuvant immunotherapy, PD-L1 staining was assessed using the 22C3 pharmDx assay (Agilent Technologies), and the CPS was calculated as the number of PD-L1-positive tumor cells, lymphocytes, and macrophages divided by the total number of viable tumor cells, multiplied by 100. A CPS of ≥1 was considered PD-L1-positive. Pathologic response was assessed using primary specimens and classified into the following categories: no pathologic response (NPR, pathologic treatment effect [PTE] < 20%), partial pathologic response (PPR, 20% ≤ PTE < 90%), major pathologic response (MPR, PTE ≥ 90%), and complete pathologic response (pCR, PTE = 100%). Image of single-stained sections were used to extract the spectrum of autofluorescence of each fluorescein, and those of the unstained sections were used to extract the spectrum of autofluorescence of tissues.

### Endpoints

The primary endpoint was the objective response rate (ORR), which was defined as the proportion of patients who achieved complete response (CR) or partial response (PR) per RECIST v1.1. Secondary endpoints included the primary lesion pCR, progression-free survival (PFS) rates (at 6, 12 and 18 months), OS rates (at 6, 12 and 18 months) and toxicities. The pCR was defined as no residual tumor tissue in primary lesion and the associated lymph nodes, along with achieving pathological remission, the PFS was defined as the time from the date of treatment initiation to the date of disease recurrence or any-cause death. The OS was defined as the time from the date of treatment initiation to the date of any-cause death. PFS is defined as the duration from the start of treatment to the first documented disease progression, recurrence, or death due to any cause. Tumor regression was independently assessed by three pathologists blinded to clinical data. Discrepancies were resolved by consensus review. Pathologic complete response (pCR) was defined as the absence of residual viable tumor cells, evaluated separately for primary tumors, lymph nodes, and combined specimens.

A consensus on MPR was reached when at least two of the three pathologists arrived at the same conclusion. Although the EORTC QLQ-HN43 is an updated version specifically designed for head and neck cancer patients, the QLQ-HN35 remains widely used in global research. In the present study, the QLQ-HN35 was therefore selected to evaluate health-related quality of life (HR-QoL) in patients who underwent surgery, as it adequately covers the major functional and symptomatic domains relevant to this population. Given the relatively small sample size, absence of prespecified HR-QoL domains, and lack of adjustment for clinical covariates, this HR-QoL analysis should be regarded as exploratory and interpreted with caution.

### Safety

The safety of the neoadjuvant therapy was determined by recording any adverse events within 30 days of receiving treatment. The adverse events were graded using the America Common Terminology Criteria for Adverse Events (CTCAE 5.0).

### Statistical analysis

The sample size calculation in this study is based on previous research and clinical experience. It is assumed that the expected ORR rate among patients with locally advanced head and neck squamous cell carcinoma receiving neoadjuvant therapy with pembrolizumab combined with chemotherapy is 60%. Meanwhile, a null hypothesis ORR rate of 40% is set, along with α level of 0.05 for the significance threshold. The binary endpoints (ORR and pCR) were presented as frequency and percentage, with corresponding 95% confidence interval (CI). The PFS and OS rates were estimated using the Kaplan-Meier curves and the corresponding 95% CIs were estimated using the Log-Log transformation method at 6-month, 12-month and 18-month follow-up.

The data of the questionnaires in 35 variables were transferred to an Excel spreadsheet and a descriptive analysis of the patients’ variables was performed to calculate the mean (standard deviation), median (interquartile range [IQR]), minimum and maximum scores of the HR-QoL scales. The HR QoL variables 1–30 were analyzed and compared between those with chemoimmunotherapy + chemoradiotherapy and those with chemoradiotherapy only, using the T-test or the Mann-Whitney U test; the rest of the variables were compared between the same groups using the Pearson’s chi-squared test or the Fisher’s exact test. The interpretation of the results is based on the correlation found only for the variables with statistical significance (*p* < 0.05).

## Results

### Patient characteristics

**Figure 1:**
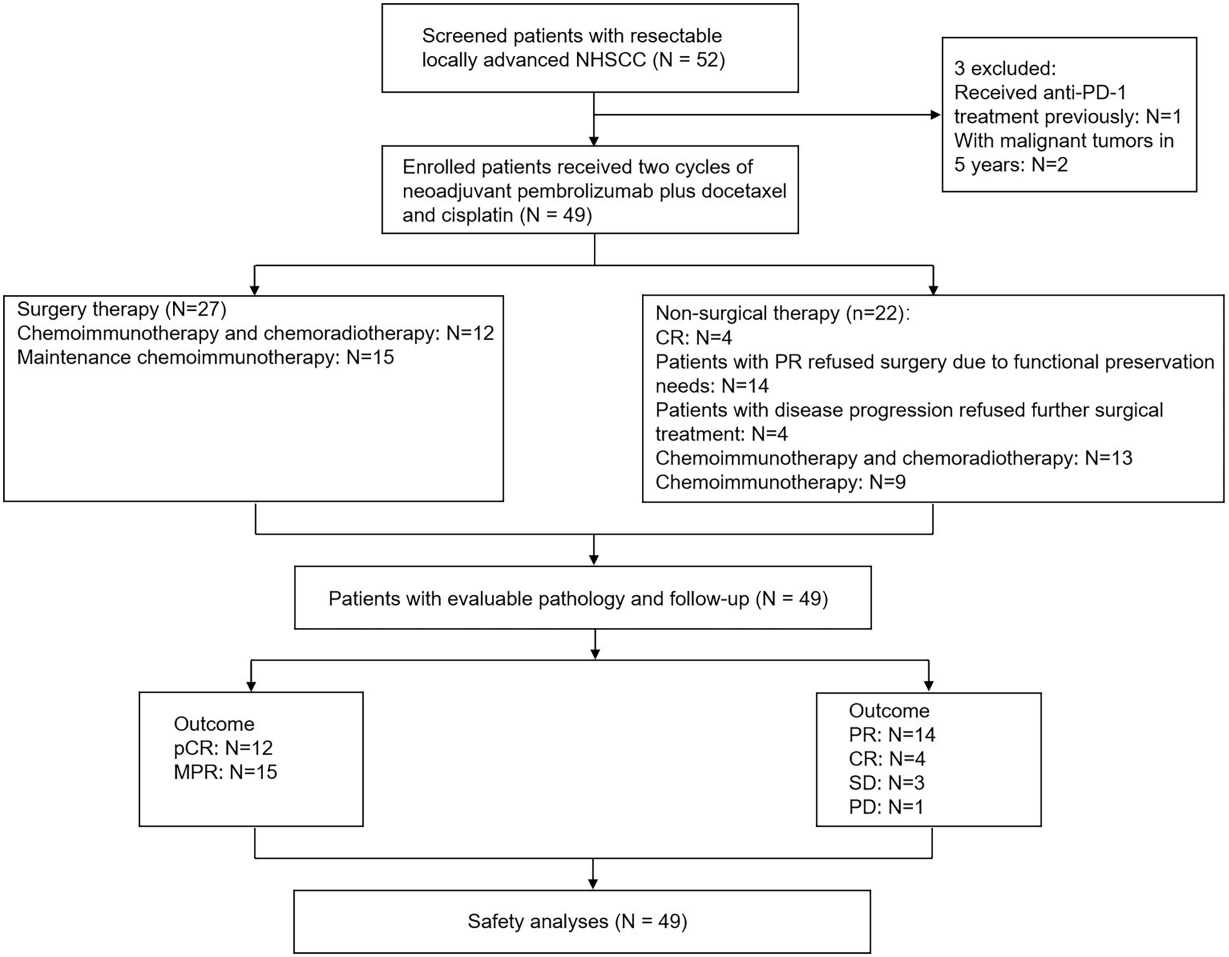
Study flow chart. NHSCC, Head and neck squamous cell carcinoma; CR, complete response; pCR, complete pathologic response; MPR, major pathologic response; PR, partial response; SD, stable disease; PD, progressive disease. In total, 52 patients were enrolled in this trial and 49 received neoadjuvant therapy; among them, 27 patients underwent surgical resection, and 22 patients received adjuvant therapy without surgery. HNSCC, head and neck squamous cell carcinoma.

Overall, 52 patients were enrolled, with 49 completed the two cycles of neoadjuvant pembrolizumab plus docetaxel and cisplatin therapy (**Figure 1**). Twenty-seven patients underwent surgical resection, while 22 patients underwent non-surgery therapy (**Figure 1**). Demographics and clinical characteristics are shown in **Table 1**. The median age of patients was 61 years (range: 49−76 years), and 42 patients were male. 40 (81.6%) patients had a smoking history, and 45 (91.8%) patients had an alcohol consumption history. Of the 49 patients, 8 (16.3%) had oral cancer, 16 (32.7%) had oropharyngeal cancer (3 patients was positive for human papillomavirus (HPV), 3 was negative for HPV, and the HPV status of the remaining 10 patients was not determined), 16 (32.7%) had hypopharyngeal cancer, and 6 (12.2%) had laryngeal carcinoma. Most patients (n=21 [42.9%]) had clinical T3 stage and 25 (51.0%) had clinical N2 stage (**Table 1**).

**Table 1:** Demographics and clinical characteristics of the patients.

Characteristics	Patients (N = 49)
Age, median (range), years	61 (49−76)
Sex, n (%)	
Male	42 (85.7)
Female	7 (14.3)
Smoking, n (%)	
No	9 (18.4)
Yes	40 (81.6)
Alcohol use, n (%)	
Never	4 (8.2)
Ever	45 (91.8)
Primary tumor, n (%)	
Oral	8 (16.3)
Oropharyngeal	16(32.7)
Hypopharyngeal	16 (32.7)
Laryngeal carcinoma	6 (12.2)
Sinus	2 (4.1)
Surface mass	1 (2.0)
Clinical T stage, n (%)	
T1	1 (2.0)
T2	17 (34.7)
T3	21 (42.9)
T4	10 (20.4)
Clinical N stage, n (%)	
N0	11 (22.4)
N1	10 (20.4)
N2	25 (51.0)
N3	3 (6.1)
CPS score, n (%)	
<1	1 (2.0)
1−19	24 (49.0)
≥20	24 (49.0)
Treatment, n (%)	
None surgery	22 (44.9)
Surgery	27 (55.1)
Adjuvant therapy, n (%)	
Radiotherapy +immunotherapy	25 (51.0)
chemoimmunotherapy	24 (49.0)

CPS, combined positive score.

### Treatment characteristics

Out of the 52 enrolled patients, three withdrew from the study for personal and economic reasons. Consequently, 49 patients completed the two cycles of neoadjuvant pembrolizumab plus docetaxel and cisplatin therapy (**Figure 1**). Twenty-seven patients underwent surgical resection, including 4 with oral cancer, 10 with oropharyngeal cancer, 9 with hypopharyngeal cancer, and 4 with laryngeal cancer. Of the 27 patients who underwent surgery, 12 received maintenance chemoimmunotherapy and chemoradiotherapy, whereas 15 received maintenance chemoimmunotherapy only. Meanwhile, 22 patients (oral: n=3; oropharyngeal: n=6; hypopharyngeal: n=8; laryngeal carcinoma: n=2; other: n=3) received non-surgical therapy, with 13 (oropharyngeal: n=4; hypopharyngeal: n=6; oral: n=2; other: n=1) receiving chemoimmunotherapy and chemoradiotherapy and 9 (oral: n=1; oropharyngeal: n=2; hypopharyngeal: n=2; laryngeal carcinoma: n=2; other: n=2) receiving chemoimmunotherapy only. Of the 22 patients, 14 patients achieved PR, 4 achieved CR, 3 achieved SD, and 1 patient a had PD after the two cycles of neoadjuvant treatment. Among the 22 patients with non-surgical therapy, the 4 with CR after two cycles of neoadjuvant treatment directly refused surgery. For the 14 patients with partial PR, although the tumors regressed, surgical assessment showed a large resection range, they strong demand for preserving organ function and refused surgery. The one patient with PD also refused further surgical treatment and requested to continue with immunotherapy combined with chemoradiotherapy.

### Efficacy

**Figure 2:**
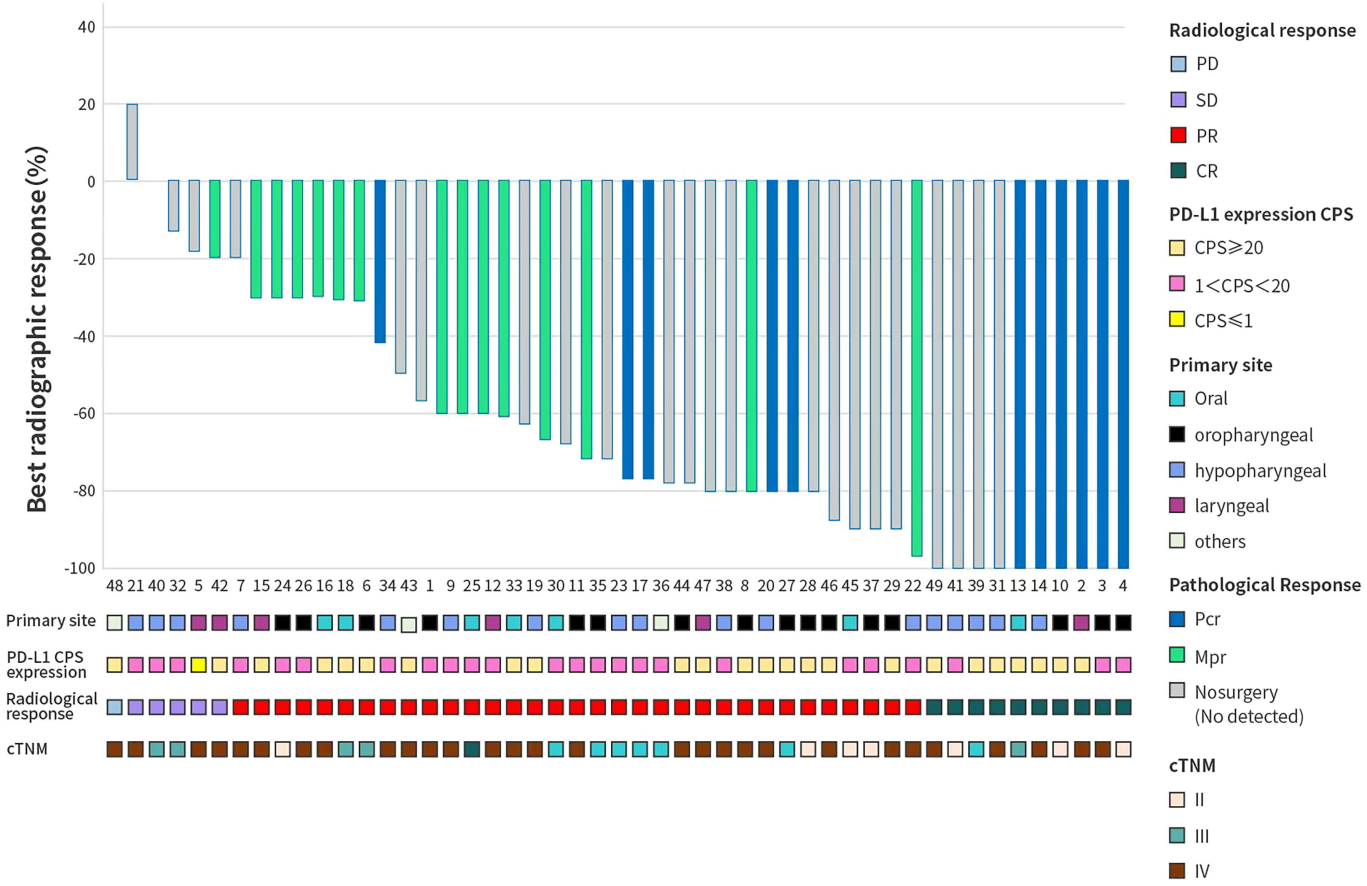
The waterfall plot of best radiographic response by RECISTv1.1, 2 weeks after the treatment with neoadjuvant pembrolizumab (200 mg) plus docetaxel (50 mg/m^2^) and cisplatin (50 mg/m^2^). Each bar indicates one patient. CPS, combined positive score; CR, complete response; MPR, major pathologic response; pCR, complete pathologic response; PD, progressive disease; PR, partial response; SD, stable disease.

After two cycles of neoadjuvant therapy, 10 patients achieved CR (oral: n=1; oropharyngeal: n=3; hypopharyngeal: n=5; laryngeal carcinoma: n=1), 33 patients achieved PR (oral: n=6; oropharyngeal: n=13; hypopharyngeal: n=9; laryngeal carcinoma: n=3; other: n=2), with an ORR of 87.8% (95% confidence interval [CI]: 75.2–95.4) (**Figure 2**). Furthermore, 1 patient had a progressive disease (PD) (laryngeal carcinoma) and 1 patient had a stable disease (SD) (neck swelling).

**Figure 3:**
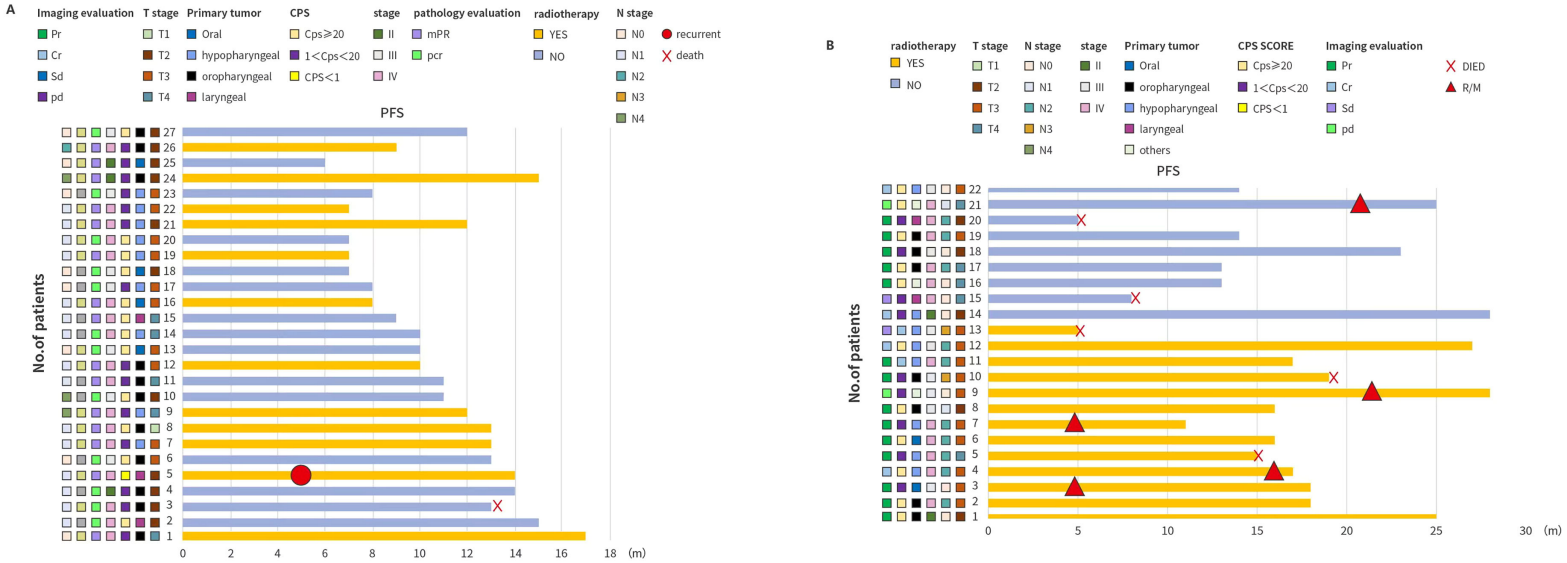
Swimming plots for **(A)** individual patients who underwent the surgical resection (n=27) and **(B)** individual patients who received adjuvant therapy without surgery (n=22). Each bar indicates one patient. CPS, combined positive score; CR, complete response; MPR, major pathologic response; pCR, complete pathologic response; PD, progressive disease; PR, partial response; SD, stable disease.

Among the 27 patients who underwent resection, 12 (44.4%, 95% CI: 25.5–64.7) achieved pCR (pre-surgery: n=6 with T3–4, and n=5 with N2, using the TNM staging system), and 15 (55.6%, 95% CI: 35.3–74.5) achieved an MPR (**Figure 3A**). Of the 12 patients who achieved a pCR, 9 patients had completely negative lymph nodes (3 had 18 negative lymph nodes, 1 had 24 negative lymph nodes and 5 had 28 negative lymph nodes) post-surgery. Similarly, out of the 15 patients who achieved MPR, 8 patients had completely negative neck lymph nodes post-surgery. Following the surgery, 12 patients who achieved MPR received chemoimmunotherapy and chemoradiotherapy, with the other 15 patients received chemoimmunotherapy only (12 patients achieved pCR and 3 achieved MPR who chose not to received radiotherapy). After 5 months, 1 patient appeared to have PD, and chose to have a resection with function preservation. One death occurred after 13 months due to cerebral infarction, which was deemed unrelated to cancer. As of 31 October 2024, there had been no metastasis, recurrence or death due to HNSCC in the surgery patient group. The core criteria for reducing the resection scope are as follows: Imaging regression: After neoadjuvant therapy, enhanced CT/MRI scans show a reduction in tumor volume of more than 50% with clear boundaries; frozen pathology during surgery indicates that no cancer cells are found in the frozen sections of the margins of the resected specimen. Functional preservation needs: Reducing the resection scope can significantly improve postoperative swallowing and phonation functions. All 27 patients had reduced resected area than initially planned, and out of the resected 13 patients with oropharyngeal, hypopharyngeal or laryngeal HNSCC, 11 (85%) patients preserved their throats.

The HR QoL assessment results for the 27 resected patients are presented in **Supplementary Table 1**. For most HR-QoL variables, patients with chemoimmunotherapy and chemoradiotherapy had higher mean H&N 35 scores than those treated with chemoimmunotherapy only, including pain in oral cavity, pain in lower jaw, mouth ulcers, pain in throat, difficulties in opening mouth, drinking, swallowing and eating, tasting, sickness. Patients with both chemoimmunotherapy and chemoradiotherapy generally felt more embarrassed of eating in front of and socializing with family and friends, compared with those with chemoimmunotherapy only.

Of the 22 unresected patients, 14 patients achieved PR, 4 achieved CR, and 3 achieved SD with one patient had a PD after the two cycles of neoadjuvant treatment (**Figure 3B**). Following which, 13 patients received chemoimmunotherapy and chemoradiotherapy, whereas 9 patients received chemoimmunotherapy only. Out of these patients, 1 had PD, and 5 died due to disease progression (**Figure 3B**).

**Figure 4:**
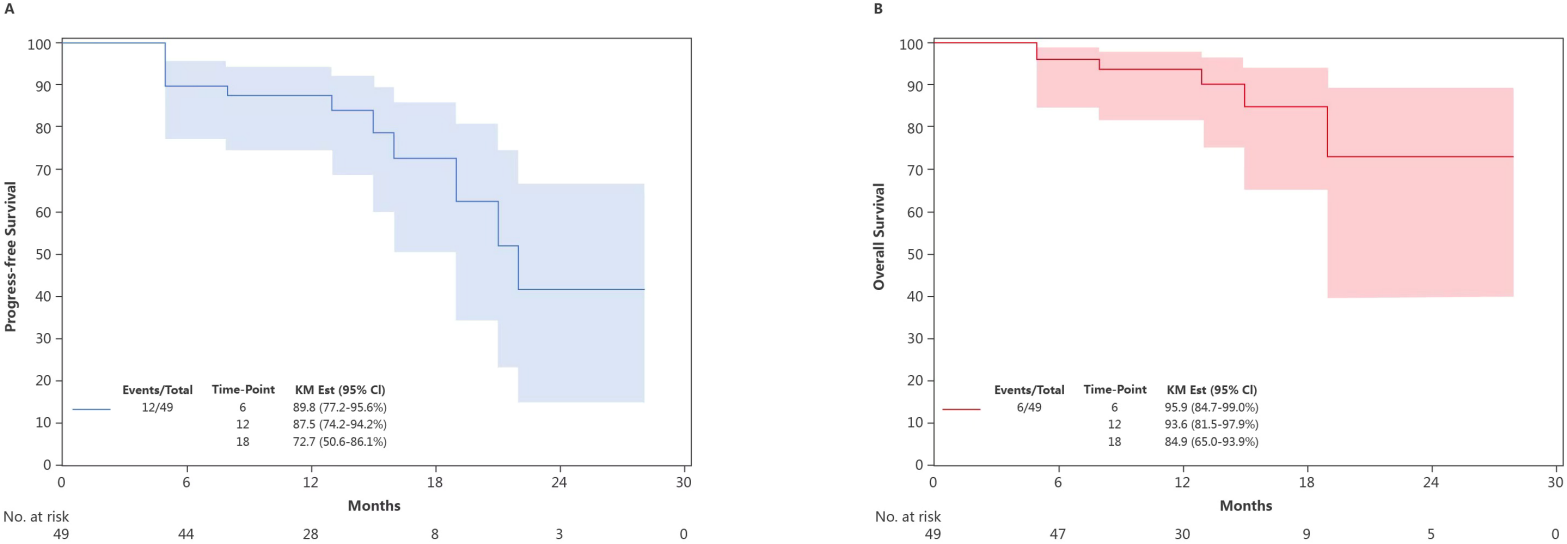
Kaplan-Meier curves depicting **(A)** PFS and **(B)** OS for patients who completed the two cycles of the neoadjuvant therapy (n=49). KM Est, Kaplan-Meier estimate; CI, confidence interval; PFS, progression-free survival; OS, overall survival.

**Figure 5:**
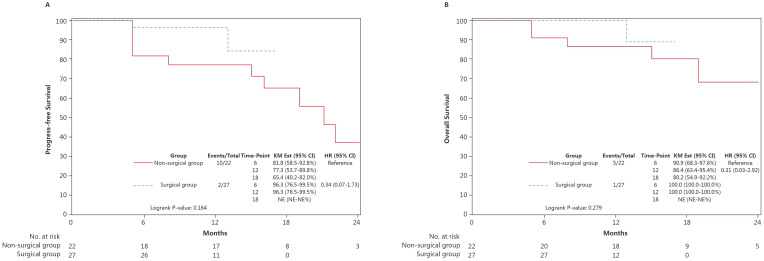
Comparison of the Kaplan-Meier curves for **(A)** PFS and **(B)** OS between the non-surgical and the surgical groups. KM Est, Kaplan-Meier estimate; CI, confidence interval; HR, hazard ratio; PFS, progression-free survival; OS, overall survival.

As of 31 October 2024, the median follow-up duration was 11 months. The estimated 6-month, 12-month and 18-month PFS rates were 89.8% (95% CI: 77.2–95.6), 87.5% (95% CI: 74.2–94.2) and 72.7% (95% CI: 50.6–86.1) (**Figure 4A**). The corresponding OS rates at 6 months, 12-months and 18 months were 95.9% (95% CI: 84.7–99.0), 93.6% (95% CI: 81.5–97.9) and 84.9% (65.0–93.9), respectively (**Figure 4B**). The surgical group had a numerically lower risk of death, compared with the non-surgical group (HR: 0.34 [0.07–1.73], p=0.164 for PFS (**Figure 5A**) and HR: 0.31 [0.03–2.92, p=0.279 for OS (**Figure 5B**)). Follow-up was still ongoing.

### Safety

**Table 2** describes the treatment-related adverse events (TRAEs). The most common TRAEs of any grades were nausea/vomiting (12.2%), fatigue (10.2%), rash (8.2%), neurotoxicity (8.2%) and elevated aminotransferases (8.2%). TRAEss of grades 3 and 4 occurred in 12 (24.5%) and 2 (4.1%) patients, respectively. There was no previously unknown or unexpected TRAEs observed. Adverse events (AEs) were summarized in all treated patients (**Table 2**).

**Table 2:** Treatment-related adverse events.

Events	Classification	Grade of adverse events		
		Grade 1−2 (n, %)	Grade 3 (n, %)	Grade 4 (n, %)
Nausea/vomiting	Chemotherapy-related	6 (12.2)	0	0
Rash	Immune-related	4 (8.2)	0	0
Fatigue	Chemotherapy-related	5 (10.2)	0	0
Leukopenia	Chemotherapy-related	0	3 (6.1)	0
Anemia	Chemotherapy-related	3 (6.1)	0	0
Neurotoxicity	Chemotherapy-related	4 (8.2)	0	0
Diarrhea	Chemotherapy-related	2 (4.1)	0	0
Pneumonia	Chemotherapy-related	2 (4.1)	3 (6.1)	0
Bleeding	Chemotherapy-related	0	1 (2.0)	1 (2.0)
aminotransferases Elevation	Chemotherapy-related	4 (8.2)	2 (4.1)	0
Creatinine Elevation	Chemotherapy-related	3 (6.1)	1 (2.0)	0
Myocarditis	Immune-related	0	0	1 (2.0)
Alopecia	Chemotherapy-related	10 (20.4)	2 (4.1)	0
Oral mucositis	Chemotherapy-related	1 (2.0)	0	0

Most treatment-related sAEs were Grade 1–2 and manageable. One patient experienced elevated creatinine levels, leading to a delay in surgery. No patients experienced severe perioperative complications or perioperative mortality.

A Grade 4 myocarditis occurred in one patient, which was considered immune-related and resolved after corticosteroid treatment. Other AEs, such as nausea, fatigue, anemia, and leukopenia, were attributed to chemotherapy toxicity. No other immune-related adverse events beyond rash and myocarditis were observed, and no patient had surgery canceled due to treatment-related toxicity.

## Discussions

In this prospective, single-arm phase II clinical trial, neoadjuvant chemotherapy immunotherapy with pembrolizumab plus docetaxel and cisplatin demonstrated promising efficacy and safety profile for resectable locally advanced HNSCC, with an ORR of 87.8% and generally manageable TRAEs. To the best of our knowledge, this is the first study to assess OS rates up to 1.5 years, which were 95.9%, 93.6% and 84.9%, at 6 months, 12-months and 18 months, respectively.

In the present study, a treatment regimen consisting of TP (docetaxel and cisplatin) without the 5-FU was employed, taking into account the toxicity with a treatment plan with TPF (13–15). Furthermore, the dose of 50 mg/m (2) for docetaxel and cisplatin was used, aiming to minimize the toxicity, thereby reducing the complication burden associated with the subsequent radiotherapy. The current used dose of 50 mg/m (2) for docetaxel and cisplatin was expected to achieve a similar efficacy as a higher dose.

The neoadjuvant pembrolizumab plus docetaxel and cisplatin showed encouraging efficacy, with a high ORR of 87.8%, further supporting the evidence with anti-PD-1 therapy for treatment of HNSCC (16, 17). This rate is consistent with the published ORR rates in previous studies in patients with HNSCC (18–20), thereby confirming that immunotherapy combined with chemotherapy is effective in improving ORR rate. In a study in patients with tumors in oropharynx, larynx and hypopharynx, receiving neoadjuvant camrelizumab and chemotherapy, an ORR of 96.7% was reported, slightly higher than that in the current study. This is probably due to the higher dose of cisplatin (75 mg/m^2^) than that in our study (50 mg/m^2^). A high ORR rate following the neoadjuvant therapy would lead to a reduced tumor burden, which is beneficial for the surgery. In the current study, the scope of the resection was initially planned based on the imaging analysis prior to the neoadjuvant therapy, but adjusted using the images following treatments. Therefore, in patients who received the surgery, the actual scope of resection was smaller than what was initially planned. Furthermore, some other patients decided to not go for the surgery mainly due to their high response to the neoadjuvant therapy. Evidently, the neoadjuvant therapy is beneficial to patients in terms of reducing the surgery burden.

In this study, a pCR rate of 44.4% (12/27) and an MPR rate of 55.6% (15/27) were achieved in the resected patients after two cycles of neoadjuvant therapy, also consistent with those reported in patients with locally advanced HNSCC (18, 21–25). In the KEYNOTE-048 study, the survival benefits of pembrolizumab monotherapy or combination chemotherapy with cetuximab in patients with recurrent or metastatic HNSCC were provided. After approximately 4 years of follow-up, it was observed that pembrolizumab monotherapy and combination chemotherapy continued to show improvements in OS, PFS, and PFS2 with subsequent treatment, supporting pembrolizumab monotherapy or combination chemotherapy as the first-line treatment for recurrent or metastatic HNSCC (7). In another open-label, multi-arm, multicenter Phase II clinical trial, the results demonstrated that the combination therapy of pembrolizumab and cetuximab exhibited encouraging clinical outcomes, with an objective response rate (ORR) as high as 45% and an overall excellent safety profile (23). It worths noting that after surgery, 17 patients achieved a large number of negative lymph nodes, indicating that the lymph nodes may be more respondent to neoadjuvant immunotherapy, compared with the primary tumor. In the group of unresected patients (n=22), 14 achieved a PR, 4 achieved a CR, 3 achieved SD with one had a PD. Notably, the surgical group had a numerically lower risk of death, compared with the non-surgical group, indicating that surgery plays a vital role in treating HNSCC along with a neoadjuvant therapy.

Despite the importance of HR-QoL of patients following chemoimmunotherapy and chemoradiotherapy (26), most studies focused primarily on efficacy and safety with treatments only. The HR QoL remains highly important in patients with HNSCCs as it can cause not only physical problems, but also psychological effects of change in appearance and loss of function. A previous study by Sharma et al, did not show significant difference in the HR QoL in patients with chemoradiotherapy compared with radiotherapy alone (27); however, another study by Klein et al, showed that combined chemoradiotherapy had a worse HR QoL compared with radiotherapy alone (28). Interestingly, the current study showed that chemoimmunotherapy and chemoradiotherapy were linked with worse HR QoL scores compared with chemoimmunotherapy alone. Further studies are warranted to analyze various factors associated with HR-QoL outcomes.

The adverse event rate in the current study was lower than that reported in a prospective phase 1/2 study with pembrolizumab plus docetaxel for the treatment of HNSCC (26). A possible reason could be the higher dose of docetaxel (75 mg/m^2^) and 6 cycles of treatment used in the above-mentioned prospective study (29).

In our study, patients who attained a pCR were administered chemoimmunotherapy and exhibited favorable therapeutic effects, with an ORR reaching 87.8%. Some experts hold the opinion that even if patients have achieved pCR still need to undergo radiotherapy to guarantee treatment efficacy and prevent potential recurrence. A meta-analysis incorporating data from randomized controlled trials indicated that for the treatment of locally advanced head and neck cancer, hyperfractionated radiotherapy with concomitant chemotherapy or induction chemotherapy with taxane, cisplatin, and fluorouracil followed by locoregional therapy more favorable outcomes compared to chemoradiotherapy (30). However, our research leans toward employing immunomaintenance therapy as an alternative to avoid the side effects potentially caused by radiotherapy. Nevertheless, given the relatively brief follow-up duration in our study, it remains inconclusive whether immunomaintenance therapy surpasses radiotherapy in terms of long-term efficacy and whether it can offer greater benefits to patients. Therefore, it is imperative to conduct longer-term follow-up to provide more sufficient evidence for treatment decision-making in patients with head and neck tumors.

The recently published phase 3 open-label randomized controlled trial, KEYNOTE-689, focused on patients with locally advanced HNSCC, with EFS as the primary endpoint. The results demonstrated that patients receiving neoadjuvant and adjuvant pembrolizumab combined with standard therapy had significantly superior EFS compared to the control group across all populations. In terms of safety, the incidence of grade 3 or higher treatment-related adverse events was similar between the two groups. In the recently announced GORTEC 2018–01 NIVOPOSTOP trial, postoperative nivolumab combined with chemoradiotherapy was compared with chemoradiotherapy alone, and a significant improvement in disease-free survival was observed (31). Our study is a prospective, single-center, single-arm, open-label phase 2 study targeting patients with resectable locally advanced HNSCC. It employed a neoadjuvant chemoimmunotherapy regimen consisting of pembrolizumab combined with docetaxel and cisplatin, after which patients could choose subsequent treatment modalities. With ORR as the primary endpoint, the study also evaluated pathological complete response rate, overall survival, and other outcomes. The results revealed a relatively high ORR, with some patients achieving pathological complete response. Our study’s neoadjuvant chemoimmunotherapy regimen of pembrolizumab combined with docetaxel and cisplatin differs from the KEYNOTE-689 regimen, which combined pembrolizumab with surgery and adjuvant chemoradiotherapy. This provides a new option for treatment regimen combinations in HNSCC, and together, these two studies offer more reference evidence for the treatment of HNSCC.

This study has several limitations. First, this is a single-arm study with no randomization, and with a relatively small sample size in a single cancer hospital, thereby with limited statistical power. However, a non-randomized subgroup analysis was performed on the non-surgical patients. Second, the follow-up time is still ongoing, and the survival outcomes at 2 years are still pending. Furthermore, the comparison in the survival outcomes between the surgical and non-surgical groups was performed for the 6- and 12-month data only, involving only 11 patients with surgery and 17 patients without surgery. Also, correlations between the radiographical and pathological responses and PFS and OS were not assessed in the current study, but are planned in future analyses when longer-term PFS and OS rates are obtained. Lastly, patients’ autonomous surgical treatment choices based on disease progression may introduce bias. Additionally, selection bias from our study’s criteria limits the generalizability of findings, and variations in healthcare providers’ skills, as well as not all patients with oropharyngeal cancer had HPV status may lead to inaccurate treatment effect estimations. Moreover, the absence of mechanistic and immunological analyses—such as tumor microenvironment or immune-cell profiling—restricts the interpretation of the underlying immunological mechanisms driving the observed clinical responses.

In conclusion, neoadjuvant chemoimmunotherapy with pembrolizumab plus docetaxel and cisplatin presented encouraging efficacy regarding radiographic and pathological anti-tumor response and an favorable safety profile. However, the mechanistic insights underlying the treatment response remain largely unexplored. A key limitation of this study, as acknowledged above, is the absence of biomarker and immunological profiling data. Incorporating such analyses in future research is of paramount importance. For instance, investigating potential biomarkers—such as PD-L1 expression levels, tumor mutational burden, or immune cell infiltration within the tumor microenvironment—could help identify patient subgroups most likely to benefit from this regimen and elucidate potential mechanisms of resistance. Furthermore, profiling dynamic changes in immune cell populations or circulating cytokines in response to therapy could provide valuable insights into the interplay between chemotherapy and immunotherapy, explaining the high response rates observed. Future studies incorporating comprehensive immunological analyses are warranted to elucidate the mechanisms of response and to further evaluate long-term survival outcomes and factors influencing patients’ HR-QoL.

## Data Availability

The raw data supporting the conclusions of this article will be made available by the authors, without undue reservation.
