# Supplementary material for: Efficacy and safety of the neoadjuvant chemoimmunotherapy with pembrolizumab plus docetaxel and cisplatin in resectable locally advanced squamous cell carcinoma of the head and neck
Source: Front Immunol. 2025 Dec 17;16:1664892. doi: 10.3389/fimmu.2025.1664892 (PMC12753483; doi:10.3389/fimmu.2025.1664892)
Supplement: Supplementary file 1 [file Table1.docx]

**Supplementary materials**

**Efficacy and safety of the neoadjuvant chemoimmunotherapy with pembrolizumab plus docetaxel and cisplatin in resectable locally advanced squamous cell carcinoma of the head and neck**

**Authors:** Jing Wang,^1^ Zhendong Li,^1^ Hui Li,^1^ Dongning Huang,^1^ Zhu Liu,^1^ Wenqian Zhang,^1^

**Affiliations:** Department of Head and Neck Surgery, Cancer Hospital of China Medical University/Liaoning Cancer Hospital and Institute

**Supplementary Table 1. HR QoL data (H&N35 scores) in patients with surgery (n=27) and those with and without chemoradiotherapy**

| **HR QoL variables** | **Patients with surgery (n=27)** | **Surgical patients with maintenance chemoimmunotherapy and chemoradiotherapy**  **(n=12)** | **Surgical patients with maintenance chemoimmunotherapy (n=15)** |
| --- | --- | --- | --- |
| 1. Pain in oral cavity | | | |
| Mean (SD) | 1.93 (0.78) | 2.42 (0.79) | 1.53 (0.52) |
| Median (IQR) | 2.00 (1.00–2.00) | 2.00 (2.00–3.00) | 2.00 (1.00–2.00) |
| Min; max | 1.00; 4.00 | 1.00; 4.00 | 1.00; 2.00 |
|  |  | z=2.89 | p=0.004 |
| 2. Pain in lower jaw | | | |
| Mean (SD) | 1.56 (0.75) | 1.92 (0.90) | 1.27 (0.46) |
| Median (IQR) | 1.00 (1.00–2.00) | 2.00(1.00~3.00) | 1.00 (1.00–2.00) |
| Min; max | 1.00; 3.00 | 1.00; 3.00 | 1.00; 2.00 |
|  |  | z=2.00 | p=0.046 |
| 3. Mouth ulcers | | | |
| Mean (SD) | 1.67 (0.83) | 2.33 (0.78) | 1.13 (0.35) |
| Median (IQR) | 1.00 (1.00–2.00) | 2.50 (2.00–3.00) | 1.00 (1.00–1.00) |
| Min; max | 1.00; 3.00 | 1.00; 3.00 | 1.00; 2.00 |
|  |  | z=3.72 | p<0.001 |
| Pain in throat | | | |
| Mean (SD) | 1.85 (0.82) | 2.42 (0.79) | 1.40 (0.51) |
| Median (IQR) | 2.00 (1.00–3.00) | 3.00 (2.00–3.00) | 1.00 (1.00–2.00) |
| Min; max | 1.00; 3.00 | 1.00; 3.00 | 1.00; 2.00 |
|  |  | z=3.10 | p=0.002 |
| Drinking difficulty | | | |
| Mean (SD) | 2.04 (0.90) | 2.67 (0.78) | 1.53 (0.64) |
| Median (IQR) | 2.00 (1.00–3.00) | 3.00 (2.00–3.00) | 1.00 (1.00–2.00) |
| Min; max | 1.00; 4.00 | 1.00; 4.00 | 1.00; 3.00 |
|  |  | z=3.24 | p=0.001 |
| Swallowing difficulty for semi-fluid diet | | | |
| Mean (SD) | 1.93 (0.78) | 2.42 (0.79) | 1.53 (0.52) |
| Median (IQR) | 2.00 (1.00–3.00) | 3.00 (2.00–3.00) | 2.00 (1.00–2.00) |
| Min; max | 1.00; 3.00 | 1.00; 3.00 | 1.00; 2.00 |
|  |  | z=2.84 | p=0.005 |
| Swallowing difficulty for solid food | | | |
| Mean (SD) | 2.00 (0.62) | 2.25 (0.62) | 1.80 (0.56) |
| Median (IQR) | 2.00 (2.00–2.00) | 2.00 (2.00–3.00) | 2.00 (1.00–2.00) |
| Min; max | 1.00; 3.00 | 1.00; 3.00 | 1.00; 3.00 |
|  |  | z=1.85 | p=0.065 |
| Swallowing obstruction | | | |
| Mean (SD) | 1.59 (0.57) | 1.83 (0.58) | 1.40 (0.51) |
| Median (IQR) | 2.00 (1.00–2.00) | 2.00 (1.50–2.00) | 1.00 (1.00–2.00) |
| Min; max | 1.00; 3.00 | 1.00; 3.00 | 1.00; 2.00 |
|  |  | z=1.89 | p=0.059 |
| Teeth issues | | | |
| Mean (SD) | 1.48 (0.70) | 1.50 (0.52) | 1.47 (0.83) |
| Median (IQR) | 1.00 (1.00–2.00) | 1.50 (1.00–2.00) | 1.00 (1.00–2.00) |
| Min; max | 1.00; 3.00 | 1.00; 2.00 | 1.00; 3.00 |
|  |  | z=0.66 | p=0.512 |
| Difficulty in opening mouth | | | |
| Mean (SD) | 1.59 (0.80) | 2.08 (0.79) | 1.20 (0.56) |
| Median (IQR) | 1.00 (1.00–2.00) | 2.00 (1.50–3.00) | 1.00 (1.00–1.00) |
| Min; max | 1.00; 3.00 | 1.00; 3.00 | 1.00; 3.00 |
|  |  | z=3.02 | p=0.003 |
| Dry mouth | | | |
| Mean (SD) | 1.59 (0.69) | 2.00 (0.60) | 1.27 (0.59) |
| Median (IQR) | 1.00 (1.00–2.00) | 2.00 (2.00–2.00) | 1.00 (1.00–1.00) |
| Min; max | 1.00; 3.00 | 1.00; 3.00 | 1.00; 3.00 |
|  |  | z=2.96 | p=0.003 |
| Viscosity of saliva | | | |
| Mean (SD) | 1.48 (0.64) | 1.92 (0.67) | 1.13 (0.35) |
| Median (IQR) | 1.00 (1.00–2.00) | 2.00 (1.50–2.00) | 1.00 (1.00–1.00) |
| Min; max | 1.00; 3.00 | 1.00; 3.00 | 1.00; 2.00 |
|  |  | z=3.20 | p=0.001 |
| Smell | | | |
| Mean (SD) | 1.41 (0.69) | 1.83 (0.83) | 1.07 (0.26) |
| Median (IQR) | 1.00 (1.00–2.00) | 2.00 (1.00–2.00) | 1.00 (1.00–1.00) |
| Min; max | 1.00; 4.00 | 1.00; 4.00 | 1.00; 2.00 |
|  |  | z=3.20 | p=0.001 |
| Taste | | | |
| Mean (SD) | 1.44 (0.51) | 1.83 (0.39) | 1.13 (0.35) |
| Median (IQR) | 1.00 (1.00–2.00) | 2.00 (2.00–2.00) | 1.00 (1.00–1.00) |
| Min; max | 1.00; 2.00 | 1.00; 2.00 | 1.00; 2.00 |
|  |  | z=3.54 | p<0.001 |
| Cough | | | |
| Mean (SD) | 1.63 (0.79) | 2.00 (0.95) | 1.33 (0.49) |
| Median (IQR) | 1.00 (1.00–2.00) | 2.00 (1.00–3.00) | 1.00 (1.00–2.00) |
| Min; max | 1.00; 3.00 | 1.00; 3.00 | 1.00; 2.00 |
|  |  | z=1.88 | p=0.061 |
| Hoarse voice | | | |
| Mean (SD) | 1.74 (0.94) | 2.17 (1.11) | 1.40 (0.63) |
| Median (IQR) | 1.00 (1.00–2.00) | 2.00 (1.00–3.00) | 1.00 (1.00–2.00) |
| Min; max | 1.00; 4.00 | 1.00; 4.00 | 1.00; 3.00 |
|  |  | z=1.95 | p=0.051 |
| Sickness | | | |
| Mean (SD) | 2.19 (0.79) | 2.67 (0.78) | 1.80 (0.56) |
| Median (IQR) | 2.00 (2.00–3.00) | 3.00 (2.00–3.00) | 2.00 (1.00–2.00) |
| Min; max | 1.00; 4.00 | 1.00; 4.00 | 1.00; 3.00 |
|  |  | z=2.90 | p=0.004 |
| Anxiety for appearance | | | |
| Mean (SD) | 1.85 (0.86) | 2.17 (0.94) | 1.60 (0.74) |
| Median (IQR) | 2.00 (1.00–2.00) | 2.00 (1.50–3.00) | 1.00 (1.00–2.00) |
| Min; max | 1.00; 4.00 | 1.00; 4.00 | 1.00; 3.00 |
|  |  | z=1.62 | p=0.106 |
| Eating difficulty | | | |
| Mean (SD) | 1.85 (0.72) | 2.33 (0.65) | 1.47 (0.52) |
| Median (IQR) | 2.00 (1.00–2.00) | 2.00 (2.00–3.00) | 1.00 (1.00–2.00) |
| Min; max | 1.00; 3.00 | 1.00; 3.00 | 1.00; 2.00 |
|  |  | z=3.05 | p=0.002 |
| Embarrassment of eating in front of family | | | |
| Mean (SD) | 1.52 (0.64) | 1.83 (0.72) | 1.27 (0.46) |
| Median (IQR) | 1.00 (1.00–2.00) | 2.00 (1.00–2.00) | 1.00 (1.00–2.00) |
| Min; max | 1.00; 3.00 | 1.00; 3.00 | 1.00; 2.00 |
|  |  | z=2.18 | p=0.029 |
| Embarrassment of eating in front of non-family | | | |
| Mean (SD) | 2.04 (0.76) | 2.50 (0.52) | 1.67 (0.72) |
| Median (IQR) | 2.00 (1.00–3.00) | 2.50 (2.00–3.00) | 2.00 (1.00–2.00) |
| Min; max | 1.00; 3.00 | 2.00; 3.00 | 1.00; 3.00 |
|  |  | z=2.80 | p=0.005 |
| Difficulty in enjoying eating | | | |
| Mean (SD) | 2.33 (0.88) | 2.83 (0.83) | 1.93 (0.70) |
| Median (IQR) | 2.00 (2.00–3.00) | 3.00 (2.50–3.00) | 2.00 (1.00–2.00) |
| Min; max | 1.00; 4.00 | 1.00; 4.00 | 1.00; 3.00 |
|  |  | z=2.69 | p=0.007 |
| Difficulty in talking to others | | | |
| Mean (SD) | 1.85 (0.82) | 2.33 (0.78) | 1.47 (0.64) |
| Median (IQR) | 2.00 (1.00–2.00) | 2.00 (2.00–3.00) | 1.00 (1.00–2.00) |
| Min; max | 1.00; 4.00 | 1.00; 4.00 | 1.00; 3.00 |
|  |  | z=2.79 | p=0.005 |
| Difficulty in talking via phone calls | | | |
| Mean (SD) | 1.96 (0.98) | 2.58 (0.79) | 1.47 (0.83) |
| Median (IQR) | 2.00 (1.00–3.00) | 3.00 (2.00–3.00) | 1.00 (1.00–2.00) |
| Min; max | 1.00; 4.00 | 1.00; 4.00 | 1.00; 4.00 |
|  |  | z=3.14 | p=0.002 |
| Difficulty in socialising with family | | | |
| Mean (SD) | 1.63 (0.56) | 1.92 (0.51) | 1.40 (0.51) |
| Median (IQR) | 2.00 (1.00–2.00) | 2.00 (2.00–2.00) | 1.00 (1.00–2.00) |
| Min; max | 1.00; 3.00 | 1.00; 3.00 | 1.00; 2.00 |
|  |  | z=2.32 | p=0.020 |
| Difficulty in socialising with friends | | | |
| Mean (SD) | 2.04 (0.76) | 2.67 (0.49) | 1.53 (0.52) |
| Median (IQR) | 2.00 (1.00–3.00) | 3.00 (2.00–3.00) | 2.00 (1.00–2.00) |
| Min; max | 1.00; 3.00 | 2.00; 3.00 | 1.00; 2.00 |
|  |  | z=3.85 | p<0.001 |
| Embarrassment in showing in public | | | |
| Mean (SD) | 1.96 (0.76) | 2.50 (0.52) | 1.53 (0.64) |
| Median (IQR) | 2.00 (1.00–3.00) | 2.50 (2.00–3.00) | 1.00 (1.00–2.00) |
| Min; max | 1.00; 3.00 | 2.00; 3.00 | 1.00; 3.00 |
|  |  | z=3.27 | p=0.001 |
| Difficulty in physical contact with family and friends | | | |
| Mean (SD) | 1.74 (0.66) | 2.08 (0.67) | 1.47 (0.52) |
| Median (IQR) | 2.00 (1.00–2.00) | 2.00 (2.00–2.50) | 1.00 (1.00–2.00) |
| Min; max | 1.00; 3.00 | 1.00; 3.00 | 1.00; 2.00 |
|  |  | z=2.33 | p=0.020 |
| Reduction in willingness for intimacy/sexual activity | | | |
| Mean (SD) | 2.78 (0.80) | 3.17 (0.72) | 2.47 (0.74) |
| Median (IQR) | 3.00 (2.00–3.00) | 3.00 (3.00–4.00) | 2.00 (2.00–3.00) |
| Min; max | 2.00; 4.00 | 2.00; 4.00 | 2.00; 4.00 |
|  |  | z=2.33 | p=0.020 |
| Difficult in enjoying intimacy/sexual activity | | | |
| Mean (SD) | 2.67 (0.92) | 3.17 (0.72) | 2.27 (0.88) |
| Median (IQR) | 3.00 (2.00–3.00) | 3.00 (3.00–4.00) | 2.00 (2.00–3.00) |
| Min; max | 1.00; 4.00 | 2.00; 4.00 | 1.00; 4.00 |
|  |  | z=2.60 | p=0.009 |
| Use of pain killer | | | |
| Yes | 26 (96.30) | 11 (91.67) | 15 (100.0) |
| No | 1 (3.70) | 1 (8.33) | 0 (0.00) |
|  |  |  | p=0.444 |
| Use of nutrients | | | |
| Yes | 27 (100.0) | 12 (100.0) | 15 (100.0) |
| No | 0 (0.00) | 0 (0.00) | 0 (0.00) |
|  |  |  | p=N/A |
| Use of nasal feeding | | | |
| Yes | 25 (92.59) | 12 (100.0) | 13 (86.67) |
| No | 2 (7.41) | 0 (0.00) | 2 (13.33) |
|  |  |  | p=0.487 |
| Weight loss | | | |
| Yes | 14 (51.85) | 9 (75.00) | 5 (33.33) |
| No | 13 (48.15) | 3 (25.00) | 10 (66.67) |
|  |  | Chi-Square=4.64 | p=0.031 |
| Weight gain | | | |
| Yes | 8 (29.63) | 2 (16.67) | 6 (40.00) |
| No | 19 (70.37) | 10 (83.33) | 9 (60.00) |
|  |  |  | p=0.236 |

HR QoL, health-related quality of life; Max, maximum; min, minimum; N/A, not applicable; SD, standard deviation;
